# Stress-induced NLRP3 inflammasome activation negatively regulates fear memory in mice

**DOI:** 10.1186/s12974-020-01842-0

**Published:** 2020-07-07

**Authors:** Yuan Dong, Shuoshuo Li, Yiming Lu, Xiaoheng Li, Yajin Liao, Zhixin Peng, Yunfeng Li, Lin Hou, Zengqiang Yuan, Jinbo Cheng

**Affiliations:** 1 Department of Biochemistry, Medical College, Qingdao University, Qingdao, 266071 Shandong China; 2 The State Key Laboratory of Brain and Cognitive Sciences, Institute of Biophysics, Chinese Academy of Sciences, Beijing, 100101 China; 3 The College of Life Sciences, University of Chinese Academy of Sciences, Beijing, 100049 China; 4 Beijing Institute of Radiation Medicine, Beijing, 100850 China; 5 The Brain Science Center, Beijing Institute of Basic Medical Sciences, No. 27 Taiping Road, Haidian District, Beijing, 100850 China; 6 Center on Translational Neuroscience, College of Life & Environmental Science, Minzu University of China, Beijing, 100081 China; 7 School of Medicine, University of South China, Hengyang, Hunan China; 8 Department of New Drug Evaluation, Beijing Institute of Pharmacology and Toxicology, Beijing, 100850 China

**Keywords:** Fear memory, PTSD, NLRP3 inflammasome, Neuroinflammation, Postsynaptic density

## Abstract

**Background:**

Persistent inflammation dysregulation and cognitive decline have been associated with several trauma- and stress-related disorders such as posttraumatic stress disorder (PTSD) and anxiety disorder. Despite the abundant discoveries of neuroinflammation in such disorders, the underlying mechanisms still remain unclear.

**Method:**

Wild-type and *Nlrp3*^*−/−*^ mice were exposed to the electric foot shocks in the contextual fear memory paradigm. Three hours after the electric foot shocks, activation of the NLRP3 inflammasome was investigated through immunoblotting and ELISA. Microglia were isolated and analyzed by quantitative real-time PCR. Hippocampal tissues were collected 3 h and 72 h after the electric foot shocks and subjected to RNA sequencing. MCC950 was administrated to mice via intraperitoneal (i.p.) injection. Interleukin-1 receptor antagonist (IL-ra) and interleukin-1β (IL-1β) were delivered via intracerebroventricular (i.c.v.) infusion. Contextual fear responses of mice were tested on 4 consecutive days (test days 1-4) starting at 48 h after the electric foot shocks. Anxiety-like behaviors were examined by elevated plus maze and open-field test.

**Results:**

We demonstrated that, in the contextual fear memory paradigm, the NLRP3 inflammasome was activated 3 h after electric foot shocks. We also found an upregulation in toll-like receptor and RIG-I-like receptor signaling, and a decrease in postsynaptic density (PSD) related proteins, such as PSD95 and Shank proteins, in the hippocampus 72 h after the electric foot shocks, indicating an association between neuroinflammation and PSD protein loss after stress encounter. Meanwhile, *Nlrp3* knockout could significantly prevent both neuroinflammation and loss of PSD-related proteins, suggesting a possible protective role of NLRP3 deletion during this process. For further studies, we demonstrated that both genetic knockout and pharmaceutical inhibition of the NLRP3 inflammasome remarkably enhanced the extinction of contextual fear memory and attenuated anxiety-like behavior caused by electric foot shocks. Moreover, cytokine IL-1β administration inhibited the extinction of contextual fear memory. Meanwhile, IL-1ra significantly enhanced the extinction of contextual fear memory and attenuated anxiety-like behavior.

**Conclusion:**

Taken together, our data revealed the pivotal role of NLRP3 inflammasome activation in the regulation of fear memory and the development of PTSD and anxiety disorder, providing a novel target for the clinical treatment of such disorders.

## Introduction

Fear memory plays a central role in the development and onset of trauma- and stress-related disorders, such as posttraumatic stress disorder (PTSD) [1]. Fear memory has been one of the most intensively studied areas and has been investigated in multiple animal models. By fear conditioning, adverse unconditioned stimuli (US) can alter the impact of neutral conditioned stimuli (CS) in certain neuronal circuits, thereby eliciting specific fear behavior induced by US. Fear conditioning is accomplished relying on the process of associative learning [2]. Meanwhile, mechanisms of fear inhibition exist. Instead of forgetting, extinction is believed to be a new learning process, during which a new association between CS and US is established. Extinction learning involves the modification of synaptic connections in neuronal circuits similar to fear conditioning [2, 3]. In contextual fear memory, the direct association between the context and US is established under bidirectional communication between amygdala and hippocampus [4–6]. The hippocampus is responsible for the contextual fear memory in the establishment and retrieval of detailed contextual representation in fear and extinction memories [7–11]. Particularly, the projections from the hippocampus to the amygdala and the medial prefrontal cortex (mPFC) are involved in the context-dependent fear response after extinction through increasing neuronal activity [12, 13].

Chronic low-grade neuroinflammation has been reported in PTSD, anxiety, and major depressive disorders (MDD) [14–16]. Clinically, multiple studies have indicated that such disorders are related to elevated circulating concentrations of pro-inflammatory cytokines, such as tumor necrosis factor-α (TNF-α), interleukin 1β (IL-1β), IL-6, and interferon-γ (IFN-γ) [16–18]. Meanwhile, altered immune responses and increased pro-inflammatory reactions are also observed in patients with PTSD [16]. Despite the abundant discoveries of neuroinflammation in PTSD and related disorders, the underlying mechanisms still remain unclear.

As pattern recognition receptors (PRRs) in the innate immune system, NACHT, LRR, and PYD domains-containing protein 3 (NLRP3) can recognize a wide range of stimuli, including Nigericin, reactive oxygen species (ROS), extracellular adenosine triphosphate (ATP), and crystalline uric acid [19]. NLRP3 inflammasome activation has been suggested a robust link to the onset and progression of a wide range of central nervous system (CNS) diseases, such as Alzheimer’s disease (AD) [20, 21], Parkinson’s disease (PD) [22–25], anxiety [26, 27], and MDD [27–30]. Importantly, previous studies showed that upon the stimulation of stress, danger-associated molecular patterns (DAMPs), such as ATP and heat shock proteins (HSPs), can induce the activation of NLRP3 inflammasome [31, 32]. NLRP3 inflammasome is tightly regulated by a two-step signaling. A priming signal occurs leading to transcriptional upregulation of NLRP3 and pro-IL-1β. Upon activation, the NLRP3 inflammasome is assembled with apoptosis-associated speck-like protein containing a CARD (ASC), leading to the maturation of interleukin-1β-converting enzyme caspase-1, which subsequently cuts pro-IL-1β into its activated form (IL-1β) [19]. In animal studies, stress exposure increases IL-1β concentrations in multiple brain regions [33]. Both insufficient and excessive levels of IL-1β impair the formation of memory [34], indicating that IL-1β is important for normal learning and memory formation. IL-1 receptor null mutant mice show enhanced fear memory and decreased anxiety behavior [35]. Meanwhile, the central administration of IL-1β can induce anxiety-like behavior and enhance fear memory after stress encounters [35, 36]. However, despite the increasing evidences linking IL-1β production, neuroinflammation, fear memory, and related disorders, it remains unclear whether NLRP3 inflammasome serves as a causal factor and how it acts on fear memory. In this study, using both genetic and pharmaceutical strategies, we characterized the important roles of NLRP3 inflammasome in the regulation of neuroinflammation in fear memory.

## Material and methods

### Mice

All mice used in this study were C57BL/6 mice, 10-week-old males, weighted around 20 g. Mice were kept under ambient photoperiod at 26 ± 1 °C, had free access to standard rodent chow and clean water. *Nlrp3* knockout mice (C57BL/6 background) were a generous gift from Prof. Rongbin Zhou (University of Science and Technology of China, Hefei, China). All animal experiments were approved by the Institutional Animal Care and Use Committee at Beijing Institute of Basic Medical Sciences.

MCC950 (Selleck, 1 mg/kg) administration was carried out via intraperitoneal (i.p.) injection for 3 consecutive days before the start of the contextual fear memory paradigm and extinction training. And then 1 h prior to each extinction train at the test days 1-3. Sterile saline i.p. injection was set as a control group.

### Contextual fear memory paradigm

Experimental procedures were performed according to Zhang et al. with modifications [37]. Generally, in the contextual fear memory paradigm, mice were introduced to the fear conditioning chambers (35 cm × 20 cm × 20 cm, Jiliang Tech) followed by a 5-min adaptation period. A total of 15 intermittent inescapable electric foot shocks (0.8 mA, 10 s with 10 s interval) were delivered. Mice in control groups were exposed to the fear conditioning chambers for an equivalent amount of time without the electric foot shocks. Fear conditioning chambers were wiped clean with 75% ethanol solution between tests. Forty-eight hours after the electric foot shocks, fear-conditioned mice were reintroduced to the same fear conditioning chambers once a day for 4 consecutive days for extinction training (test days 1-4). Mice in the control group were placed to the same fear conditioning chambers for an equivalent amount of time. Spontaneous activity (5 min) was recorded during extinction training. Freezing behavior of mice associated with contextual fear memory induced by adverse experience [37]. Percentages of cumulative freezing time during spontaneous activity were used to reflect the fear responses of mice. All mice were tested throughout the procedure. Spontaneous activities were recorded and analyzed by Jiliang Tech analysis system.

### Elevated plus maze

Elevated plus maze (EPM) contains 2 open arms (35 × 5 cm) and 2 enclosed arms (35 × 5 cm) connected by a center area (5 × 5 cm). The apparatus was lifted up 50 cm above the floor. Tests were carried out under a quiet and dimly lit environment. The apparatus was wiped clean with 75% ethanol between tests. Mice were introduced to EPM 24 h after the end of the extinction procedure. Mice were placed in the center area gently, facing to one of the open arms. Spontaneous activities were monitored for a 5-min period. Number of entries, time spent, and distance traveled in the open arms by the mice were analyzed by the ANY-maze software (Global Biotech).

### Open-field test

Open-field (OF) apparatus (50 × 50 × 20 cm) was placed in a quiet and dimly lit environment, wiped clean with a 75% ethanol solution between tests. Twenty-four hours after EPM, mice were introduced to OF. Spontaneous activities were monitored for a 5-min period. Number of entries, time spent, and distance traveled in the center area (25 × 25 cm) by mice were analyzed by the ANY-maze software.

### Stereotaxic surgery

Mice were anesthetized by pentobarbital sodium (70 mg/kg, dissolved in saline) via i.p. injection and immobilized on the stereotaxic apparatus (RWD). A guide cannula (OD 0.41 mm, C = 2.2 mm, RWD) was stereotaxically positioned into the lateral ventricle at the following coordinates from bregma: AP, −0.4 mm; ML, 1 mm; DV, −2.2 mm. The guide cannula was secured to the skull with dental cement and steel screws (M1.0 × L2.0 mm, RWD). Cap (OD 0.2, G = 0.5 mm, RWD) was screwed into guide cannula. Mice were maintained individually in cage and allowed to recover for 7 days before the start of infusion.

### Intracerebroventricular infusion

The delivery system contains an injector cannula (OD 0.21 mm, C = 4 mm, G = 0.5 mm, RWD) fixed on a polyethylene (PE50) tube (OD 0.85 mm, RWD, filled with mineral oil) and connected to a syringe microinjector (5 μl, Hamilton). The infusion procedure was conducted 30 min before the start of the contextual fear memory paradigm and each extinction training. Mice were kept at the state of being conscious and able to move freely during infusion. Cap was removed before infusion. Injector cannula was inserted into guide cannula, secured on by fixing screws (OD 5.5 mm). The infusion procedure was programmed at the rate of 0.1 μl/min (total volume 1 μl) and delivered by a micro flow rate syringe pump (Longer Pump). After each infusion, the injector cannula was remained in the guide cannula for 10 min. Recombinant mouse IL-1 receptor antagonist (IL-1ra, R&D Systems, dissolved in sterile saline) was infused at a dose of 90 μg/kg each time at test days 0-3. This dose of IL-1ra was chosen according to previous researches proving it was able to block depressive-like behavior in mice [38–40]. Recombinant mouse IL-1β (R&D Systems, dissolved in sterile saline) was infused at a dose of 1 ng/kg each time (test days 0-3). Same volume of sterile saline was infused as control.

### Microglia isolation

Mice were anesthetized by pentobarbital sodium via i.p. injection and cardiac perfused with saline. Microglia were isolated according to the procedure described previously [41, 42]. Whole-brain tissue was freshly harvested, cut into small pieces, suspended in Dounce buffer (1.5 mM HEPES, 0.5% glucose in HBSS buffer), and homogenized gently by Dounce homogenizer. Homogenates of brain tissue were suspended in phosphate-buffered saline (PBS; 8 g/l NaCl, 0.2 g/l KCl, 1.44 g/l Na2HPO4, 0.24 g/l KH2PO4), filtered with cell strainers (70 μm), and centrifuged at 600×*g* for 6 min (4 °C) to collect the cell pellets. One hundred percent of percoll solution was prepared with an absolute percoll (GE Healthcare) dissolved in 10× PBS (9:1), and further diluted (v/v) to 70%, 37%, and 30% with PBS. Cell pellets were suspended in 37% percoll solution. Microglia were isolated by density gradient centrifugation. Density gradient was added into 15 ml centrifuge tubes, by layers of percoll solution from bottom to top containing: 70%, 37% (with cell suspension), 30% percoll solution, and PBS. Centrifuge was carried out in a horizontal centrifuge at 2000×*g* for 30 min (4 °C). Microglia were converged on the interphase between 37% and 70% percoll solution. Isolated microglia were washed with 10× volumes of PBS, and centrifuged at 600×*g* for 6 min (4 °C). Cell pellets were ready for mRNA extraction.

### Tissue harvesting

Mice were anesthetized by pentobarbital sodium via i.p. injection and cardiac perfused with saline. Hippocampal tissue was harvested freshly from the brain and stored at −80 °C until use.

### Quantitative real-time PCR

Total RNA of hippocampal tissue was extracted by Trizol regent (Invitrogen). Total RNA of isolated microglia was extracted by NucleoSpin RNA Plus XS kit (Macherey-Nagel). A 0.5 μg aliquot of total RNA of each sample was reversely transcribed using a one-step first strain cDNA synthesis kit (Transgen). Primer sequences used for quantitative real-time PCR (qPCR) were listed in Table S1. qPCR reactions were performed on QuantStudio 3 (Applied Biosystems) using 2× SYBR Green PCR master mix (Genestar). Data were quantified with comparative *C*_t_ method (2^−ΔΔCt^) based on *C*_t_ values normalized to β-actin. All tests were performed in triplicates.

### Immunoblot analysis

Samples were prepared in SDS loading buffer (50 mM Tris HCl, 2% SDS, 0.1% bromophenol blue, 10% glycerol, 1% β-mercaptoethanol, pH = 7.0), fractionated by SDS-PAGE and transferred to nitrocellulose film (GE Healthcare). The film was then blocked in 5% skimmed milk. Following, primary and secondary antibodies were used for immunoblotting: NLRP3 (AdipoGen, AG-20B-0014, 1:1000), Caspase-1 (AdipoGen, Ag-20B-0042, 1:1000), PSD95 (Cell Signaling, 2507, 1:1000), Shank2 (Absin, abs134803, 1:1000), Shank3 (Cell Signaling Technology, 64555, 1:1000), β-actin (Invitrogen, MA5-11869, 1:3000), Goat anti-rabbit IgG (Jackson ImmunoResearch Laboratories, 1:5000), Goat anti-mouse IgG (Jackson ImmunoResearch Laboratories, 1:5000). Immunoblot was visualized by ECL (Thermo Scientific) and analyzed using software Image J.

### Immunohistochemistry and immunofluorescence

All procedures were performed as previously described [43, 44]. Briefly, mice were anesthetized by pentobarbital sodium and perfused with saline. Brains were fixed with 4% paraformaldehyde (w/v) for 7 days. Fixed brains were transferred to 30% sucrose solution for 3 days. Coronal sections were cut throughout the whole brain. In immunohistochemistry (IHC), brain slices were blocked with 10% goat serum (Abcom, ab7481) in PBS containing 0.2% Triton X-100 (Sigma, V900502), and incubated with Iba1 primary antibody (WAKO, 019-19741, 1:400), biotinylated goat anti-rabbit IgG, and streptavidin-conjugated horseradish peroxidase using VECTASTAIN® ABC-HRP kit (Vector Laboratories, PK-4000). Iba1 stains were visualized with 3,3′-diaminobenzidine (Sigma-Aldrich), scanned and analyzed by stereo investigator (MicroBrightfield). In immunofluorescence, brain slices were blocked, and incubated with primary and secondary antibodies as follow: Iba1 (Novus Biologicals, NB100-1028, 1:400), ASC (Cell Signaling Technology, D2W8U, 1:500), Alexa Fluor 546-conjugated (Invitrogen, A-11056, 1:400), and FITC-conjugated (Abcam, ab6798, 1:400). Hoechst 33258 (Sigma) were incubated for the visualization of nuclear morphology.

### ELISA

Mouse IL-1β was measured in hippocampal homogenates according to the manufacturer’s instructions (R&D Systems). Hippocampal homogenates were prepared in cell lysis buffer (50 mM Hepes pH 7.4, 150 mM NaCl, 1% Nonidet P-40, 0.1% deoxycholate, 0.05% SDS, 0.1 M NaF, 1 mM EGTA, and protease inhibitor cocktail), and aligned according to protein concentrations measured by Pierce™ BCA Protein Assay kit (Thermo Scientific). All tests were performed in triplicates.

### RNA sequencing and analysis

mRNA of hippocampal tissue was extracted and purified by the Dynabeads mRNA purification kit (Invitrogen, 61006). Aliquots of mRNA extracted from five mice were mixed in each group and fragmented for cDNA library constructing. cDNA library was sequenced by Illumina HiSeq 4000 sequencing platform. Low-quality reads in the raw data were trimmed using Trimmomatic v0.33 [45]. Clean data were mapped against Mus musculus reference genome (GRCm38) by STAR, then read counts were used for the expression level quantification of each gene using RSEM [46]. The differentially expressed genes (fold change > 1 and the adjust *p* value < 0.05) between two groups were analyzed by DESeq v1.10.1 (adjust *p* < 0.05) [47]. Pathway and process enrichment analysis was conducted by Metascape (http://metascape.org/gp/index.html) [48]. Gene Set Enrichment Analysis (GSEA) from the target passway was performed using the GSEA v2.0.14 software (http://www.broadinstitute.org/gsea/index.jsp). Heat-map representation of gene expression was generated by the “pheatmap” package of R (https://CRAN.R-project.org/package = pheatmap).

### Statistical analysis

All results were expressed as mean ± SEM. Statistical analysis was performed by GraphPad Prism 6. Data were subjected to *t* tests (unpaired *t* test with Welch’s correction) or ANOVA for significance of difference, to where appropriate. A significant level was set to *p* < 0.05 for all statistical analysis.

## Results

### Electric foot shock exposure in the contextual fear paradigm induces anxiety-like behavior

Freezing behavior is associated with contextual fear memory induced by electric foot shock, which has been widely used in studies of fear memory and extinction [37]. Here, we found that electric foot shock exposure induced the elevation of contextual fear response (Fig. S1a-b; *t* test, *p* < 0.01). Moreover, contextual fear response declined after extinction training (Fig. S1c; analysis of variance [ANOVA]: *F*_(3, 28)_ = 7.33, *p* < 0.01), demonstrating the extinction of conditioned fear memory. *T* test was also conducted at each time point, revealing a significant lower fear response at test days 3 and 4 than the freezing level at test day 1 (Fig. S1c; *t* test, *p* < 0.05). In the EPM test, we found that fear-conditioned mice displayed significant lower entries, time spent, and distance traveled at open arms than control mice (Fig. S1d-g). In the OF test, fear-conditioned mice showed significant lower entries, time spent, and distance traveled at center area than control mice (Fig. S1h-k).

### The NLRP3 inflammasome is activated in electric foot shock-induced contextual fear memory

**Fig. 1:**
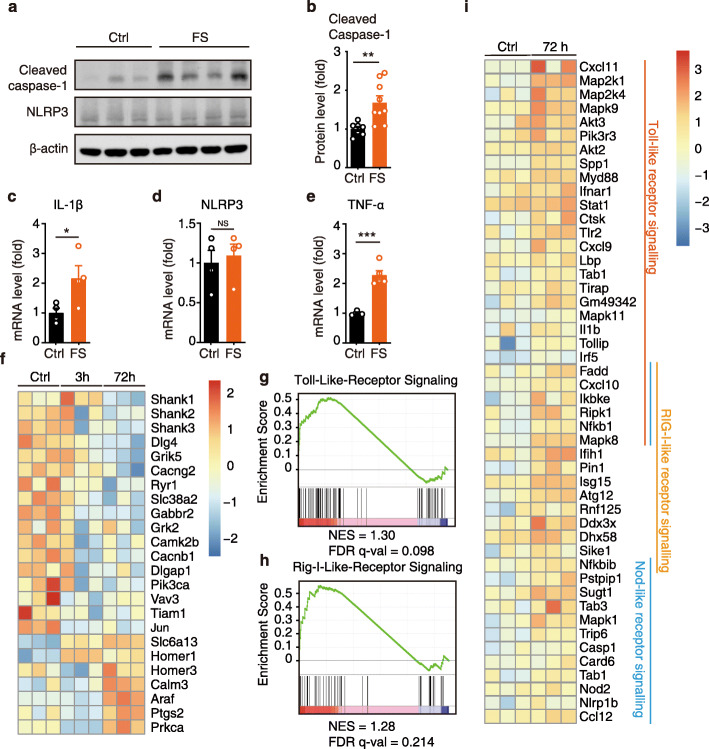
NLRP3 inflammasome is activated in electric foot shock induced contextual fear memory. **a**, **b** Representative image of immunoblotting and quantitative analysis of cleaved caspase-1, NLRP3, and β-actin in the hippocampus collected from control mice (no electric foot shock) and fear conditioned mice at 3 h post the electric foot shocks. Control (Ctrl), *n* = 7; electric foot shocks (FS), *n* = 9. **c**-**e** qPCR analysis of IL-1β, NLRP3, and TNF-α mRNA levels in isolated microglia collected from control and fear conditioned mice at 3 h post the electric foot shocks. **c**, **d***n* = 4 per group. **e** Ctrl, *n* = 3; FS, *n* = 5. **f** Heatmap of differential expression genes related to neuronal functions in hippocampus collected from control mice and fear conditioned mice at 3 h and 72 h post electric foot shocks. **g**, **h** GSEA of toll-like receptor signaling and RIG-I-like receptor signaling, FS vs Ctrl. **i** Heatmap of differential expression genes related to inflammation at 72 h post the electric foot shocks. Data shown are mean ± SEM. Data were analyzed by *t* test (**b**-**e**). NS, no significance, *p < 0.05, **p < 0.01, ***p < 0.001

We investigated the involvement of the NLRP3 inflammasome in electric foot shock-induced contextual fear memory. Mice were exposed to electric foot shocks in the fear conditioning chambers. As the hippocampus plays important roles in the regulation of contextual fear response, we selected this brain region for further tests. The hippocampal tissue was collected 3 h after the electric foot shocks and prepared for immunoblotting. We found that cleaved caspase-1 levels were significantly elevated in the hippocampus 3 h after the electric foot shocks, indicating the activation of the NLRP3 inflammasome (Fig. 1a, b). Likewise, immunofluorescence staining revealed ASC specks formation 3 h after electric foot shocks, colocalizing with microglia (Fig. S2a). However, we found unchanged NLRP3 protein levels (Fig. 1a) and no significant changes in either *Il-1β* or *Nlrp3* mRNA levels in the hippocampal tissue 3 h after the electric foot shocks (Fig. S2b-c). Microglia, which are considered as the residential immune cells of the CNS, play central roles in the regulation of neuroinflammation. Therefore, microglial cells were isolated from the whole-brain tissue of the mice. The results revealed increased *Il-1β* and *Tnf-α* mRNA levels, but not *Nlrp3*, in the isolated microglia 3 h after exposure to electric foot shocks (Fig. 1c-e). The above results suggested the activation of NLRP3 inflammasome shortly after the stress encounter. However, the activation level was relatively low and probably restricted within the microglia.

For further analyses, genome-wide differential expression of hippocampal tissue collected from control mice and fear-conditioned mice at 3 h and 72 h after the electric foot shocks were conducted. Pathway and process enrichment analysis revealed the top 20 significantly up- and downregulated pathways (Fig. S2d-e). Several pathways related to synapse and neuronal function were significantly downregulated 72 h after the electric foot shocks. Among the significantly altered genes, postsynaptic proteins, including *discs large MAGUK scaffold protein 4* (*Dlg4*, encoding gene for postsynaptic density protein 95 [PSD95]), *SH3*, *and multiple ankyrin repeat domains* 1 (*Shank1*)*, Shank2*, and *Shank3* significantly decreased 72 h after the electric foot shocks (Fig. 1f). Meanwhile, GSEA also revealed a significant upregulation of genes related to toll-like receptor ( NES = 1.30, FDR *q* val = 0.098, FWER *p* val = 0.00), and RIG-I-like receptor (NES = 1.28, FDR *q* val = 0.214, FWER *p* val = 0.056) signaling pathways 72 h after the electric foot shock (Fig. 1g-i, Fig. S2f). Taken together, these results indicate that exposure to electric foot shock in contextual fear memory induces the activation of NLRP3 inflammasome and the upregulation of neuroinflammation.

### *Nlrp3* knockout attenuates transcriptional changes in neuroinflammation and postsynaptic proteins in contextual fear memory

**Fig. 2:**
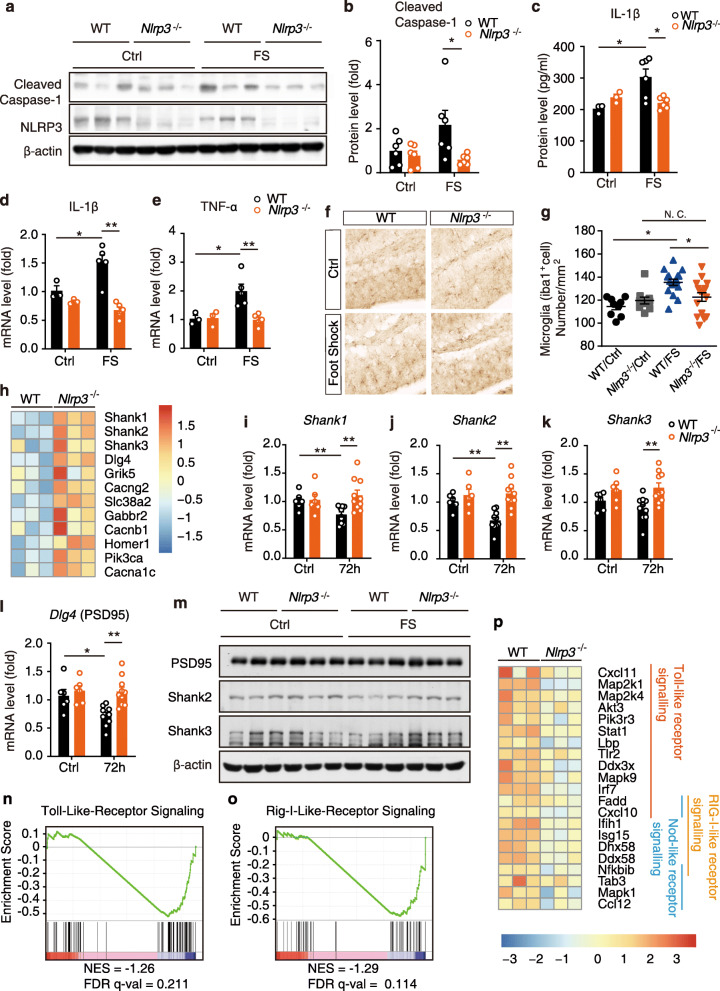
*Nlrp3* knockout attenuated transcriptional changes in neuroinflammation and postsynaptic proteins in contextual fear memory. **a**, **b** Representative images of immunoblotting and quantitative analysis of cleaved caspase-1, NLRP3, and β-actin in the hippocampus collected from WT and *Nlrp3*^*−/−*^ mice 3 h post the electric foot shocks. *n* = 6 in each group. **c** ELISA of IL-1β level in hippocampus 3 h post the electric foot shocks. WT/Ctrl, *n* = 3; *Nlrp3*^*−/−*^/Ctrl, *n* = 3; WT/FS, *n* = 5; *Nlrp3*^*−/−*^/FS, *n* = 5. **d**, **e** qPCR analysis of IL-1β and TNF-α mRNA levels in isolated microglia collected from WT and *Nlrp3*^*−/−*^ mice 3 h post the electric foot shocks. WT/Ctrl, *n* = 3; *Nlrp3*^*−/−*^/Ctrl, *n* = 3; WT/FS, *n* = 5; *Nlrp3*^*−/−*^/FS, *n* = 5. **f**, **g** Immunohistochemistry and statistical analysis of Iba1^+^ cells in hippocampus. **h** Heatmap of differential expression genes related to neuronal functions in hippocampus collected from WT and *Nlrp3*^*−/−*^ mice 72 h post electric foot shocks. **i**-**l** qPCR analysis of Shank1, Shank2, Shank3, and PSD95 (*Dlg4*) mRNA level in WT and *Nlrp3*^*−/−*^ mice 72 h after the electric foot shocks. WT/Ctrl, *n* = 6; *Nlrp3*^*−/−*^/Ctrl, *n* = 6; WT/FS, *n* = 10; *Nlrp3*^*−/−*^/FS, *n* = 10. **m** Immunoblotting of PSD95, Shank2, and Shank3 protein levels in WT and *Nlrp3*^*−/−*^ mice 72 h after the electric foot shocks. **n**, **o** GSEA of toll-like receptor signaling and RIG-I-like receptor signaling. *Nlrp3*^*−/−*^ vs WT. **p** Heatmap of differential expression genes related to inflammation at 72 h after the electric foot shocks. Data shown are mean ± SEM. Data were analyzed by *t* test. NS, no significance, *p < 0.05, **p < 0.01

Previous sections of this study suggested the activation of the NLRP3 inflammasome, and transcriptional changes related to postsynaptic proteins and neuroinflammation in electric foot shock-induced contextual fear memory. However, the causal linkages among them remain unclear. *Nlrp3*^−/−^ mice were introduced to electric foot shocks in fear conditioning chambers. Immunoblotting of the hippocampal samples collected 3 h after the electric foot shock revealed that *Nlrp3* knockout inhibited the elevation of cleaved caspase-1 levels (Fig. 2a, b). Additionally, a significant increase in IL-1β levels in the hippocampus of WT mice 3 h after the electric foot shocks was revealed by ELISA, while *Nlrp3* knockout significantly inhibited this increase (Fig. 2c). In isolated microglia, *Nlrp3* knockout dramatically inhibited the increase in *Il-1β* and *Tnf-α* mRNA levels (Fig. 2d, e). Consistent with the results, we found increased microglial (Iba1^+^) cell numbers in the hippocampus 72 h after exposure to electric foot shocks, suggesting a possible prolonged effect caused by the activation of NLRP3 inflammasome induced by stress encounter. Meanwhile *Nlrp3*^−/−^ mice displayed significant lower Iba1^+^ cell numbers than that in WT mice, indicating a lower activation level of microglia (Fig. 2f, g).

Genome-wide differential expression in RNA-sequencing and pathway and process enrichment analysis revealed the top 20 significantly upregulated and downregulated pathways (Fig. S3a-b). Compared with WT mice, *Nlrp3*^*−/−*^ mice exhibited significant higher transcriptional levels in several pathways related to synapse and neuronal function and lower transcriptional levels in pathways related to immune response 72 h after the electric foot shocks. Higher levels of *Dlg4*, *Shank1*, *Shank2*, *and Shank3* mRNA expressions were observed in *Nlrp3*^−/−^ mice than in WT mice 72 h after the electric foot shocks (Fig. 2h, Fig. S3c). This result was confirmed by qPCR. In particular, significant decreases in *Dlg4*, *Shank1*, and *Shank2* mRNA levels were observed in fear-conditioned mice 72 h after the electric foot shocks in WT mice (Fig. 2i-l). Meanwhile, *Nlrp3*^−/−^ mice had significant higher levels of *Dlg4*, *Shank1*, *Shank2*, *and Shank3* mRNA 72 h after the electric foot shocks than did the WT mice (Fig. 2i-l). The protein levels of PSD95, Shank2, and Shank3 were also confirmed by immunoblotting (Fig. 2m, Fig. Sd-f). Downward trends of PSD95, Shank2, and Shank3 were detected 72 h after the electric foot shocks in WT mice. Compared with WT mice, *Nlrp3*^−/−^ mice expressed significant higher levels of PSD95, Shank2, and Shank3 proteins 72 h after the electric foot shocks. Moreover, *Nlrp3*^−/−^ mice expressed the downregulation of both toll-like receptor (NES = −1.26, FDR *q* val = 0.211, FWER *p* val = 0.057) and RIG-I-like receptor (NES = −1.29, FDR *q* val = 0.114, FWER *p* val = 0.00) signaling compared with WT mice (Fig. 2n-p). Overall, we found that *Nlrp3* knockout in mice not only abolished the activation of NLRP3 inflammasome induced by electric foot shock in contextual fear memory but also alleviated the decrease in postsynaptic proteins and inhibited the upregulation of neuroinflammation, suggesting that the NLRP3 inflammasome may play a causal role in the electric foot shock-induced neuroinflammation and loss of postsynaptic proteins.

### *Nlrp3* knockout enhances fear extinction and attenuates anxiety-like behavior

**Fig. 3:**
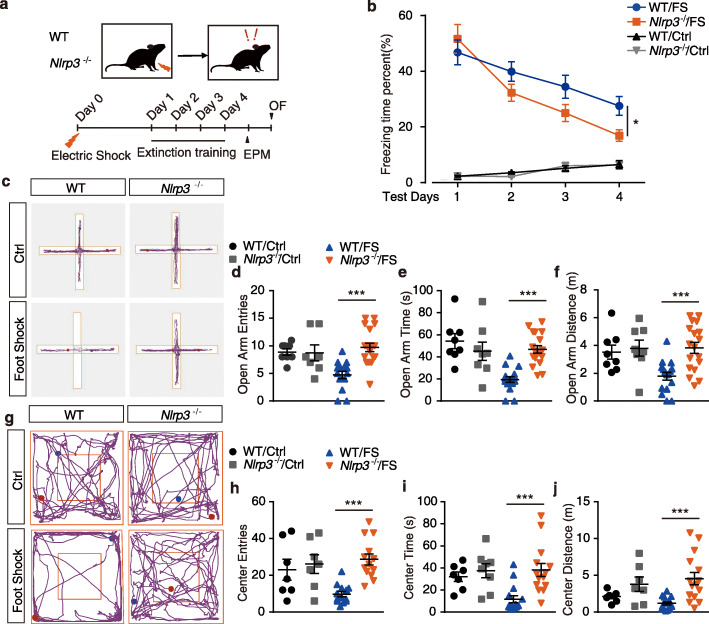
*Nlrp3* knockout enhances fear extinction and attenuates anxiety-like behavior. **a** Trial schematic for contextual fear memory paradigm, extinction training, and behavior tests. **b** Fear responses of 4 extinction training days. WT/Ctrl, *n* = 6; *Nlrp3*^*−/−*^/Ctrl, *n* = 6; WT/FS, *n* = 12; *Nlrp3*^*−/−*^/FS, *n* = 12. **c** Representative image of mice track plot in EPM test. **d**-**f** EPM test after 4 extinction trainings. WT/Ctrl, *n* = 7; *Nlrp3*^*−/−*^/Ctrl, *n* = 7; WT/FS, *n* = 15; *Nlrp3*^*−/−*^/FS, *n* = 15. **g** Representative image of mice track plot in OF test. **h-j** OF test after 4 extinction trainings. WT/Ctrl, *n* = 7; *Nlrp3*^*−/−*^/Ctrl, *n* = 7; WT/FS, *n* = 15; *Nlrp3*^*−/−*^/FS, *n* = 15. Data shown are mean ± SEM. Data were analyzed by one-way ANOVA (**b**), two-way ANOVA (**b**), and *t* test (**d**-**f**, **h**-**j**). NS, no significance; *p < 0.05; **p < 0.01; ***p < 0.001

To further study the function of the NLRP3 inflammasome in contextual fear memory, WT and *Nlrp3*^−/−^ mice were introduced to the contextual fear memory paradigm and extinction training, as shown in Fig. 3a. In the normal condition (without foot shocks), *Nlrp3* knockout mice displayed unaltered (Fig. 3b; ANOVA: *F*_(1, 36)_ = 0.05, *p* = 0.82) fear responses compared with WT mice. However, electric foot shock exposure significantly elevated contextual fear responses in both WT and *Nlrp3*^−/−^ mice on test day 1 (Fig. 3b and Fig. S4a), indicating that the knockout of *Nlrp3* did not impair the process of contextual fear memory acquisition. Two-way ANOVA conducted on the fear responses throughout the four extinction training days revealed a significantly enhanced fear memory extinction in *Nlrp3*^−/−^ mice (extinction, *F*_(3, 84)_ = 19.01, *p* < 0.01; gene, *F*_(1, 84)_ = 4.701, *p* < 0.05; interaction, *F*_(3,84)_ = 1.889, *p* = 0.138). Moreover, on test day 4 (after 3 extinction trainings), *Nlrp3*^−/−^ mice displayed a significant lower fear response than WT mice (Fig. S4b; *t* test, *p* < 0.05). In EPM test, no significant difference in the anxiety behavior was detected at the normal condition (no foot shock) between WT and *Nlrp3*^−/−^ mice. Notably, *Nlrp3*^−/−^ mice displayed significant attenuated anxiety-like behavioral preference induced by foot shocks, as evidenced by significant higher entries, time spent, and distance traveled at open arms compared with WT mice (Fig. 3c-f; *t* test, *p* < 0.01). In the OF test, *Nlrp3*^-/-^ mice displayed significantly attenuated anxiety-like behavioral preference induced by foot shock, as evidenced by significant higher entries, time spent, and distance traveled at the center area observed in knockout mice than in WT mice (Fig. 3g-j; *t* test, *p* < 0.01).

### Administration of MCC950 enhances fear extinction and attenuates anxiety-like behavior

**Fig. 4:**
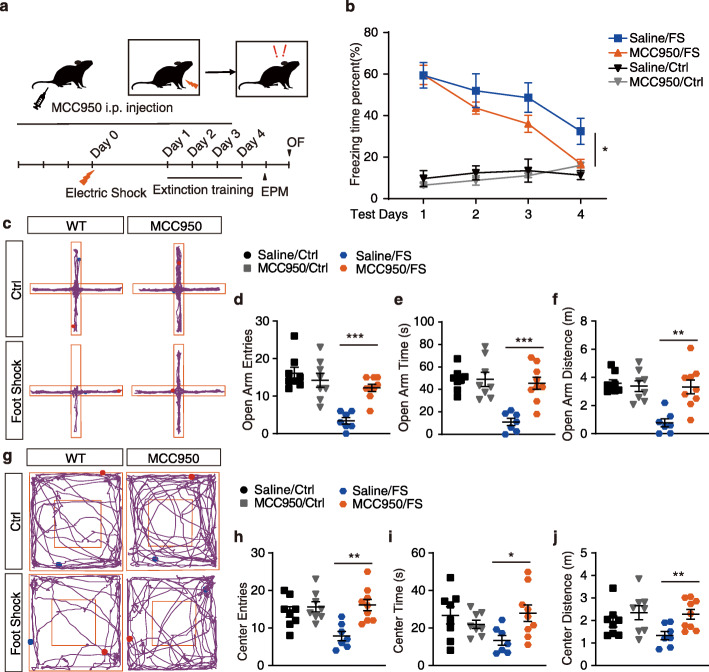
Administration of MCC950 enhances fear extinction and attenuates anxiety-like behavior. **a** Trial schematic for MCC950 administration (1 mg/kg), contextual fear memory paradigm, extinction training, and behavior tests. **b** Fear responses of 4 extinction training days. Saline/Ctrl, *n* = 4; MCC950/Ctrl, *n* = 4; saline/FS, *n* = 7; MCC950/FS, *n* = 12. **c** Representative image of mice track plot in EPM test. **d-f** EPM test after 4 extinction trainings. Saline/Ctrl, *n* = 8; MCC950/Ctrl, *n* = 8; saline/FS, *n* = 7; MCC950/FS, *n* = 9. **g** Representative image of mice track plot in OF test. **h-j** OF test after 4 extinction trainings. Saline/Ctrl, *n* = 8; MCC950/Ctrl, *n* = 8; saline/FS, *n* = 7; MCC950/FS, *n* = 9. Data shown are mean ± SEM. Data were analyzed by one-way ANOVA (**b**), two-way ANOVA (**b**), and *t* test (**d**-**f**, **h**-**j**). NS, no significance; *p < 0.05; **p < 0.01; ***p < 0.001

To further confirm the function of NLRP3 inflammasome activation in contextual fear memory, MCC950, a selective inhibitor of the NLRP3 inflammasome [49, 50], was administrated to mice, as shown in Fig. 4a. Consistent with the results in *Nlrp3*^−*/*−^*mice*, treatment of MCC950 showed no effect in mice under normal conditions (without foot shocks) (Fig. 4b; ANOVA: *F*_(1, 24)_ = 0.27, *p* = 0.61). Moreover, a similar level of contextual fear response was induced by electric foot shocks in both saline- and MCC950-recipient mice (Fig. 4b, Fig. S4c, *t* test, *p* = 0.978). However, two-way ANOVA on the fear responses throughout the four extinction training days revealed a significantly enhanced fear memory extinction in MCC950-recipient mice (extinction, *F*_(3, 61)_ = 14.26, *p* < 0.01; MCC950, *F*_(1, 61)_ = 6.194, *p* < 0.05; interaction, *F*_(3,61)_ = 0.815, *p* = 0.49). On test day 4 (after 3 extinction trainings), MCC950-recipient mice displayed significant lower fear response than saline-recipient mice (Fig. S4d; *t* test, *p* < 0.05). In the EPM and OF tests, MCC950 treatment significantly rescued the behavioral preference induced by foot shock compared with saline, as evidenced by significant higher entries, time spent, and distance traveled at the open arms and center area, respectively (Fig. 4c-j). Meanwhile, no significant difference in the anxiety-like behavior was detected at the normal condition between MCC950- and saline-recipient mice. The above results demonstrate that the inhibition of the activation of the NLRP3 inflammasome also enhances the process of fear memory extinction and attenuates anxiety-like behavior induced by electric foot shock in contextual fear memory.

### The NLRP3 inflammasome downstream cytokine interleukin-1β is involved in fear extinction

**Fig. 5:**
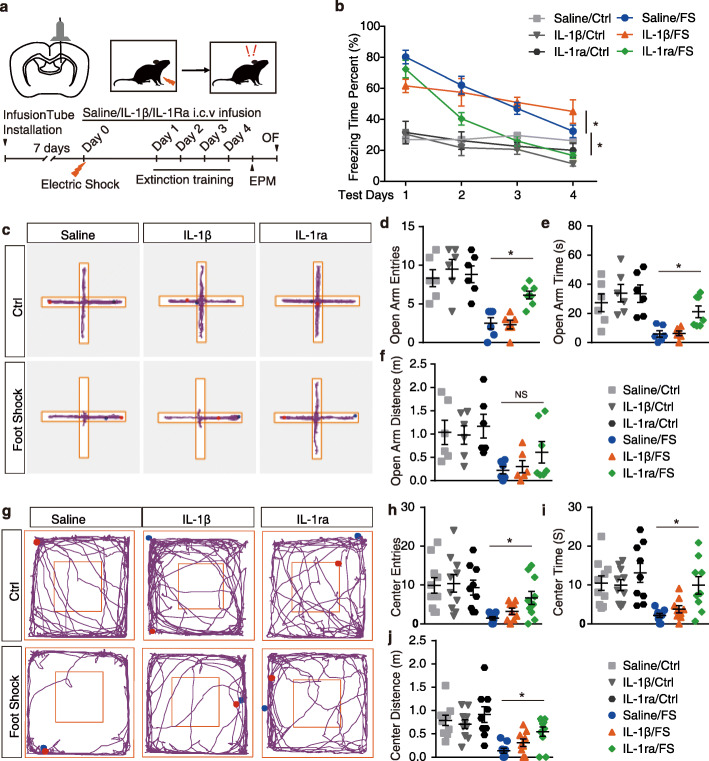
The NLRP3 inflammasome downstream cytokine IL-1β is involved in fear extinction. **a** Trial schematic for IL-1β (1 ng/kg) and IL-1ra (90 μg/kg) administration, contextual fear memory paradigm, extinction training, and behavior tests. **b** Fear responses of 4 extinction training days. Saline/Ctrl, *n* = 4; IL-1β/Ctrl, *n* = 4; IL-1ra/Ctrl, *n* = 6; saline/FS, *n* = 12; IL-1β/FS, *n* = 12; IL-1ra/FS, *n* = 12. **c** Representative image of mice track plot in EPM test. **d-f** EPM test after 4 extinction trainings. Saline/Ctrl, *n* = 6; IL-1β/Ctrl, *n* = 6; IL-1ra/Ctrl, *n* = 6; saline/FS, *n* = 6; IL-1β/FS, *n* = 6; IL-1ra/FS, *n* = 7. **g** Representative image of mice track plot in OF test. **h-j** OF test after 4 extinction trainings. Saline/Ctrl, *n* = 10; IL-1β/Ctrl, *n* = 10; IL-1ra/Ctrl, *n* = 9; saline/FS, *n* = 9; IL-1β/FS, *n* = 9; IL-1ra/FS, *n* = 10. Data shown are mean ± SEM. Data were analyzed by one-way ANOVA (**b**), two-way ANOVA (**b**) and *t* test (**d**-**f**, **h**-**j**). NS, no significance; *p < 0.05; **p < 0.01; ***p < 0.001

Our results demonstrate that both genetic knockout and pharmaceutical inhibition of NLRP3 inflammasome remarkably enhance extinction memory and attenuate anxiety-like behaviors. Together with the observation that IL-1β levels were increased in contextual fear memory, we questioned whether the effect of NLRP3 inflammasome on fear memory was accomplished through the alternation of downstream IL-1β signaling. To address this question, mice receiving continuous i.c.v. infusion of mouse recombinant IL-1β (1 ng/kg), IL-1ra (90 μg/kg) or saline were exposed to electric foot shocks in the contextual fear paradigm (Fig. 5a). As shown in Fig. 5b, exposure to electric foot shocks induced the elevation of the contextual fear response. Interestingly, IL-1β-recipient mice showed significant lower contextual fear responses than saline-recipient mice at test day 1 (Fig. 5b, Fig. S4e, *t* test, *p* < 0.05). Furthermore, two-way ANOVA of the fear responses during the four extinction training days revealed a differential change in fear responses of IL-1β recipient mice compared with saline (Fig. 5b; extinction, *F*_(3, 71)_ = 13.01, *p* < 0.01; treatment, *F*_(1, 71)_ = 0.167, *p* = 0.68; interaction, *F*_(3,71)_ = 3.078, *p* < 0.05). One-way ANOVA of the fear response of IL-1β recipient mice through extinction training was conducted revealing an insignificant change in fear responses, suggesting impaired fear memory extinction in IL-1β-recipient mice (Fig. 5b; *F*_(3, 31)_ = 1.29, *p* = 0.29). On test day 4, the contextual fear responses in IL-1β-recipient mice were significant higher than that in the saline-recipient mice (Fig. 5b, Fig. S4h, *t* test, *p* < 0.05), suggesting that the administration of IL-1β impaired fear memory acquisition and extinction. Meanwhile, IL-1ra-recipient mice displayed the same level of contextual fear responses with saline recipient mice on test day 1 (Fig. 5b, Fig. S4e, *t* test, *p* > 0.05). Two-way ANOVA of the fear responses during the four extinction training days revealed enhanced fear memory extinction in the IL-1ra-recipient mice than in the saline-recipient mice (Fig. 5b; extinction, *F*_(3, 81)_ = 51.98, *p* < 0.01; treatment, *F*_(1, 81)_ = 28.80, *p* < 0.01; interaction, *F*_(3,81)_ = 1.13, *p* = 0.34). Moreover, the IL-1ra-recipient mice displayed significant lower contextual fear responses than saline-recipient mice after only one extinction training (Fig. 5b; Fig. S4f-h, test days 2-4, *t* test, *p* < 0.05). In the EPM test after extinction training, IL-1ra infusion significantly rescued the anxiety-like behavioral preference compared with saline-recipient mice, evidenced by higher entries and time spent at open arms (Fig. 5c-f; *t* test, *p* < 0.05). Consistently, in the OF test, IL-1ra infusion significantly rescued the anxiety-like behavioral preference induced by foot shock in the contextual fear paradigm, as evidenced by higher entries, time spent, and distance traveled at the center area (Fig. 5g-j; *t* test, *p* < 0.05). However, compared with saline-recipient mice, the IL-1β infusion showed no effect on the anxiety-like behavior by displaying the same level of entries, time spent, and distance traveled at open arms or center area in the EPM or OF test, respectively (Fig. 5c-j). Taken together, these results suggest that antagonizing the IL-1 receptor by IL-1ra was able to regulate the fear memory probably through the enhancement of fear memory extinction. Meanwhile, agonizing the IL-1 receptor by IL-1β damaged both fear memory acquisition and extinction.

**Fig. 6:**
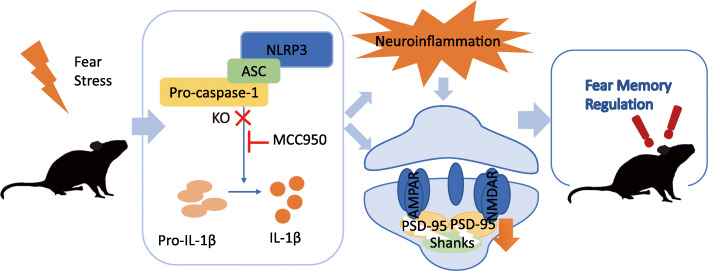
General overview of the main highlights of this study. In contextual fear paradigm, NLRP3 inflammasome is activated after electric foot shocks, causing the upregulation of neuroinflammation and decrease of PSD-related proteins, leading to the modification in fear memory regulation

In summary, our results demonstrate that the exposure to electric foot shocks in contextual fear memory induces the activation of the NLRP3 inflammasome and upregulation of neuroinflammation. Genetic knockout of *Nlrp3*, pharmaceutical inhibition of NLRP3 inflammasome, or IL-1 receptor antagonizing enhanced the extinction of fear memory and attenuated anxiety-like behaviors (Fig. 6).

## Discussions

In this study, we demonstrated that stress exposure by electric foot shocks can significantly increase the hippocampal concentration of cleaved caspase-1 and IL-1β in 3 h, suggesting an immediate activation of the NLRP3 inflammasome shortly after the stress encounter. Previous studies have found that the NLRP3 inflammasome is activated in stress-induced depressive animal models and MDD patients [28], and that antidepressant treatments inhibited it through autophagy [51]. In accordance with these findings, our results revealed the activation of the NLRP3 inflammasome in an electric food shocks-induced animal model of PTSD. Most importantly, the activation of the NLRP3 inflammasome was observed earlier than the appearance of systematic inflammation was. In the CNS, the expression of NLRP3 can be detected in microglia, astrocytes, and neurons during severe pathological conditions such as spinal cord injury [52], but can only be found in the microglia/macrophages under physiological conditions [53, 54]. A previous study has reported that the expression of NLRP3 is restricted to microglia in a study of animal models of depressive disorder [55]. In isolated microglia, we detected the transcriptional upregulation of *il-1β* and *tnf-α* 3 h after the electric foot shocks, indicating that the process of initiating neuroinflammation after stress stimulation may predominately occur in microglia. However, we cannot exclude the possible effects of peripheral immunity during this process, as the animals we used in our study were *Nlrp3* knockout and MCC950 i.p. injected mice. The activation of the NLRP3 inflammasome in mononuclear blood cells has also been reported in patients with MDD [56]. Additionally, a recent study has revealed the effect of peripheral CD4^+^ T cells in stress-induced anxiety-like behavior in mice [57]. Despite the early reaction of the NLRP3 inflammasome, microglia were activated 72 h after the electric foot shocks, as revealed by the significant increase in Iba1^+^ cell numbers in the hippocampus. This observation was also supported by GSEA, where the significant upregulation of both toll-like receptor and RIG-I-like receptor signaling occurred at 72 h after the electric foot shocks, suggesting that neuroinflammation continually developed after the stress encounter. Abnormalities in microglia can directly cause neuropsychiatric disorders in mice. Mutation of Homeobox B8 (Hoxb8, only expresses in the microglia in mice) in mice causes pathological grooming, hyperanxiety, and social impairment deficits, which are similar to the obsessive-compulsive disorder (OCD) and autism spectrum disorders (ASDs) observed in human [58, 59]. Concurrently, the loss of PSD proteins, including PSD95 and Shank proteins, suggested impaired neuronal function, which was associated with the development of neuroinflammation. Interestingly, defection in Shank proteins has been intensively studied as one of the most important causes of ASDs.

PSD95 (coded by *Dlg4*) belongs to the membrane-associated guanylate kinase (MAGUK) family and is able to interact with N-methyl-D-aspartate (NMDA) receptors, α-amino-3-hydroxy-5-methyl-4-isoxazolepropionic acid (AMPA) receptors and potassium channels at the postsynaptic site [60–62], and plays important roles in synaptic plasticity and synaptic stabilization in long-term potentiation (LTP) [63]. *Dlg4*^*−/−*^ mice display abnormalities in multiple behavior tests, including increased responses related to stress and anxiety [64]. Shank proteins are scaffold proteins predominantly localized within the PSD of glutamatergic synapses in the CNS. Direct association of Shank2 and PSD95 has been reported [65]. Shank proteins also interact with AMPA receptors, NMDA receptors, and mGLu receptors and have been associated with ASDs and other neurological diseases in the CNS [66]. Moreover, Shank3 insufficiency in the anterior cingulate cortex (ACC) can generate dysfunctions in excitatory synaptic and social interaction deficits in mice [67]. On the molecular level of fear memory, NMDA receptor-dependent neuron excitability is necessary for this process. Antagonizing or enhancing the NMDA receptor can modulate the processes for both fear conditioning and extinction [68–70]. Activation of the NDMA receptor in the hippocampus is essential for the formation and trace of contextually regulated fear memory [71]. In the hippocampus, the NMDA receptor is upregulated during extinction training in a rat model of PTSD [72]. NMDA receptor agonist D-cycloserine administration eliminates the upregulation of NMDA receptor subunits and alleviates impaired fear extinction memory [72]. Simultaneously, fear conditioning induces the trafficking of AMPA receptors in the lateral amygdala (LA), thereby affecting associative learning [73]. Importantly, it has been reported that the pro-inflammatory cytokine IL-1β can regulate the trafficking of AMPA receptors, leading to depression-like behaviors in mice after chronic social defeat stress (CSDS) [38]. In *Nlrp3* knockout mice, stress exposure induced a level of fear response (without extinction) similar to that of WT mice, suggesting an unscathed associative learning. Most remarkably, both alleviated neuroinflammation and PSD proteins lost were observed in the *Nlrp3* knockout mice 72 h after electric foot shocks, suggesting a direct impact of neuroinflammation on the integrity of neuronal functions. Improved extinction learning in *Nlrp3* knockout mice has been further proven in the extinction training procedure, as revealed by the significant lower fear response and less anxiety-like behaviors. Both MCC950 treatment and IL-1ra administration further confirmed this phenomenon by displaying similar improvements during the extinction training.

As an important form of learning and memory, extinction is essential for the regulation of fear memory. In the clinic, PTSD is often treated by exposure therapy, which is supported by extinction learning [74]. However, extinction learning deficits have been implicated in PTSD and other related neuropsychiatric disorders [75]. In addition to psychological interventions, most PTSD patients also receive pharmacologic treatments. Drugs that alleviate specific symptoms (e.g., insomnia, nightmares, and alcohol abuse) have also been recommended in the treatment of PTSD. However, these agents are usually symptoms-based and rarely induce remission. The relapse of the symptoms often occurs upon discontinuation of the agents [1]. MCC950 acts as a selective inhibitor of the NLRP3 inflammasome by targeting its ATP-hydrolysis motif [49, 50] and has been investigated to treat a wide range of CNS diseases related to the NLRP3 inflammasome [20]. The protective effects of genetic knockout and pharmaceutical inhibition of the NLRP3 inflammasome and IL1-ra suggest a novel strategy in the treatment of PTSD and related neuropsychiatric disorders. Our study provides evidence for the development of new treatments for PTSD by targeting the NLRP3 inflammasome. Further studies are required for the clinical treatment of PTSD and related disorders.

## Conclusions

In conclusion, the NLRP3 inflammasome is activated after electric foot shocks in contextual fear paradigm, followed by the upregulation of toll-like receptor and RIG-I-like receptor signaling, and a decrease in PSD-related proteins in the hippocampus 72 h after the electric foot shocks. *Nlrp3* knockout can prevent both neuroinflammation and loss of PSD-related proteins. Both genetic knockout and pharmaceutical inhibition of the NLRP3 inflammasome enhance the extinction of contextual fear memory and attenuate anxiety-like behavior caused by electric foot shocks. Our findings suggest an association between NLRP3 activation, neuroinflammation, and PSD protein loss in fear memory, providing a novel target for the treatment of trauma- and stress-related disorders, such as PTSD.

## Supplementary information

**Additional file 1.**

**Additional file 2.**

## Data Availability

Not applicable.

## Abbreviations

AD: Alzheimer’s disease
AMPA: α-amino-3-hydroxy-5-methyl-4-isoxazolepropionic acid
ASC: Apoptosis-associated speck-like protein containing a CARD
ASDs: Autism spectrum disorders
ATP: Adenosine triphosphate
BLA: Basolateral amygdala
CNS: Central nervous system
CS: Conditioned stimulus
CSDS: Chronic social defeat stress
Ctrl: Control
DAMPs: Danger-associated molecular patterns
Dlg4: Discs Large MAGUK Scaffold Protein 4
EPM: Elevated plus maze
FDA: Food and Drug Administration
FS: Electric foot shocks
Hoxb8: Homeobox B8
HSPs: Heat-shock proteins
i.c.v.: Intracerebroventricular
i.p.: Intraperitoneal
IFN-γ: Interferon-γ
IHC: Immunohistochemistry
IL-1β: Interleukin 1β
LTP: Long-term potentiation
MAGUK: Membrane-associated guanylate kinase
MDD: Major depression disorder
mPFC: Medial prefrontal cortex
NLRP3: NACHT, LRR, and PYD domains-containing protein 3
NMDA: N-methyl-D-aspartate
OCD: Obsessive-compulsive disorder
OF: Open-field
PD: Parkinson’s disease
PSD: Postsynaptic density
PSD95: Postsynaptic density protein 95
PTSD: Posttraumatic stress disorder
qPCR: Quantitative real-time PCR
Shank: SH3 And Multiple Ankyrin Repeat Domains
TNF-α: Tumor necrosis factor-α
US: Unconditioned stimulus
