# Supplementary material for: Stress-induced NLRP3 inflammasome activation negatively regulates fear memory in mice
Source: J Neuroinflammation. 2020 Jul 7;17:205. doi: 10.1186/s12974-020-01842-0 (PMC7341659; doi:10.1186/s12974-020-01842-0)
Supplement: Supplementary file 1 — Additional file 1. [file 12974_2020_1842_MOESM1_ESM.docx]

**Supplementary Table**

**Table S1** Primer sequences for qPCR

| **Primer** | **Primer type** | **Sequence 5’-3’** |
| --- | --- | --- |
| *Mouse-Il-1β* | Forward | TGTAATGAAAGACGGCACACC |
|  | Reverse | TCTTCTTTGGGTATTGCTTGG |
| *Mouse-Tnf-α* | Forward | CAGGCGGTGCCTATGTCT |
|  | Reverse | CGATCACCCCGAAGTTCAGTAG |
| *Mouse-Nlrp3* | Forward | ATTACCCGCCCGAGAAAGG |
|  | Reverse | TCGCAGCAAAGATCCACACAG |
| *Mouse-Dlg4* | Forward | TGAGATCAGTCATAGCAGCTACT |
|  | Reverse | CTTCCTCCCCTAGCAGGTCC |
| *Mouse-Shank1* | Forward | TGCATCAGACGAAATGCCTAC |
|  | Reverse | AACAGTCCATAGTTCAGCACG |
| *Mouse-Shank2* | Forward | AGAGGCCCCAGCTTATTCCAA |
|  | Reverse | CAGGGGTATAGCTTCCAAGGC |
| *Mouse-Shank3* | Forward | ATGGGCCTGTGTGGTAGTCTT |
|  | Reverse | CCACCTTATCTGTGCTGTGTAG |
| *Mouse-β-actin* | Forward | GGCTGTATTCCCCTCCATCG |
|  | Reverse | CCAGTTGGTAACAATGCCATGT |
