# Supplementary material for: Stress-induced NLRP3 inflammasome activation negatively regulates fear memory in mice
Source: J Neuroinflammation. 2020 Jul 7;17:205. doi: 10.1186/s12974-020-01842-0 (PMC7341659; doi:10.1186/s12974-020-01842-0)
Supplement: Supplementary file 2 — Additional file 2. [file 12974_2020_1842_MOESM2_ESM.pdf]

Figure S1

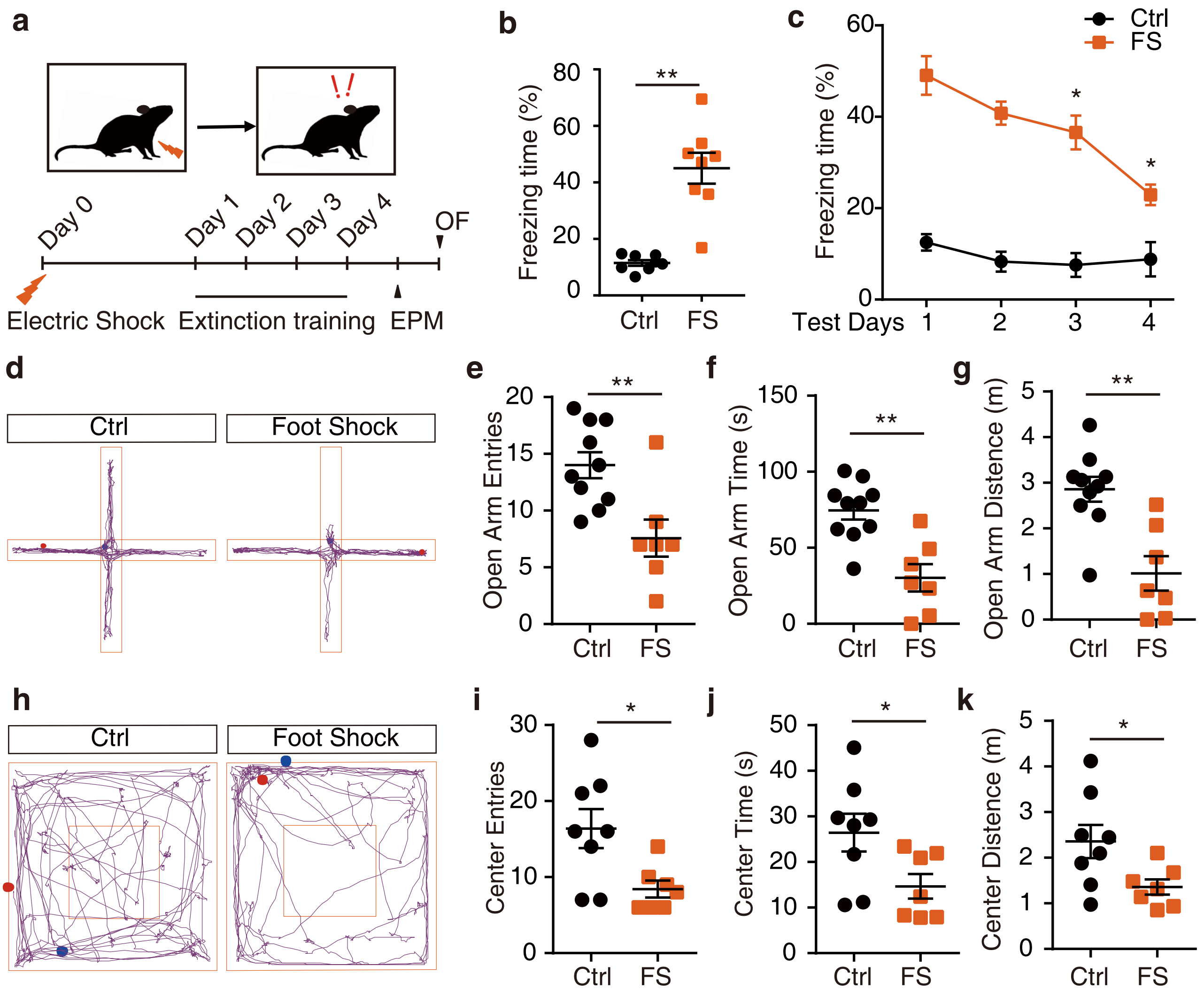

a
Day 0
Day 1
Day 2
Day 3
Day 4
OF
Electric Shock
Extinction training
EPM
b
Freezing time (%)
Ctrl
FS
c
Ctrl
FS
Freezing time (%)
Test Days
d
Ctrl
Foot Shock
e
Open Arm Entries
f
Open Arm Time (s)
g
Open Arm Distence (m)
h
Ctrl
Foot Shock
i
Center Entries
j
Center Time (s)
k
Center Distence (m)

Figure S2

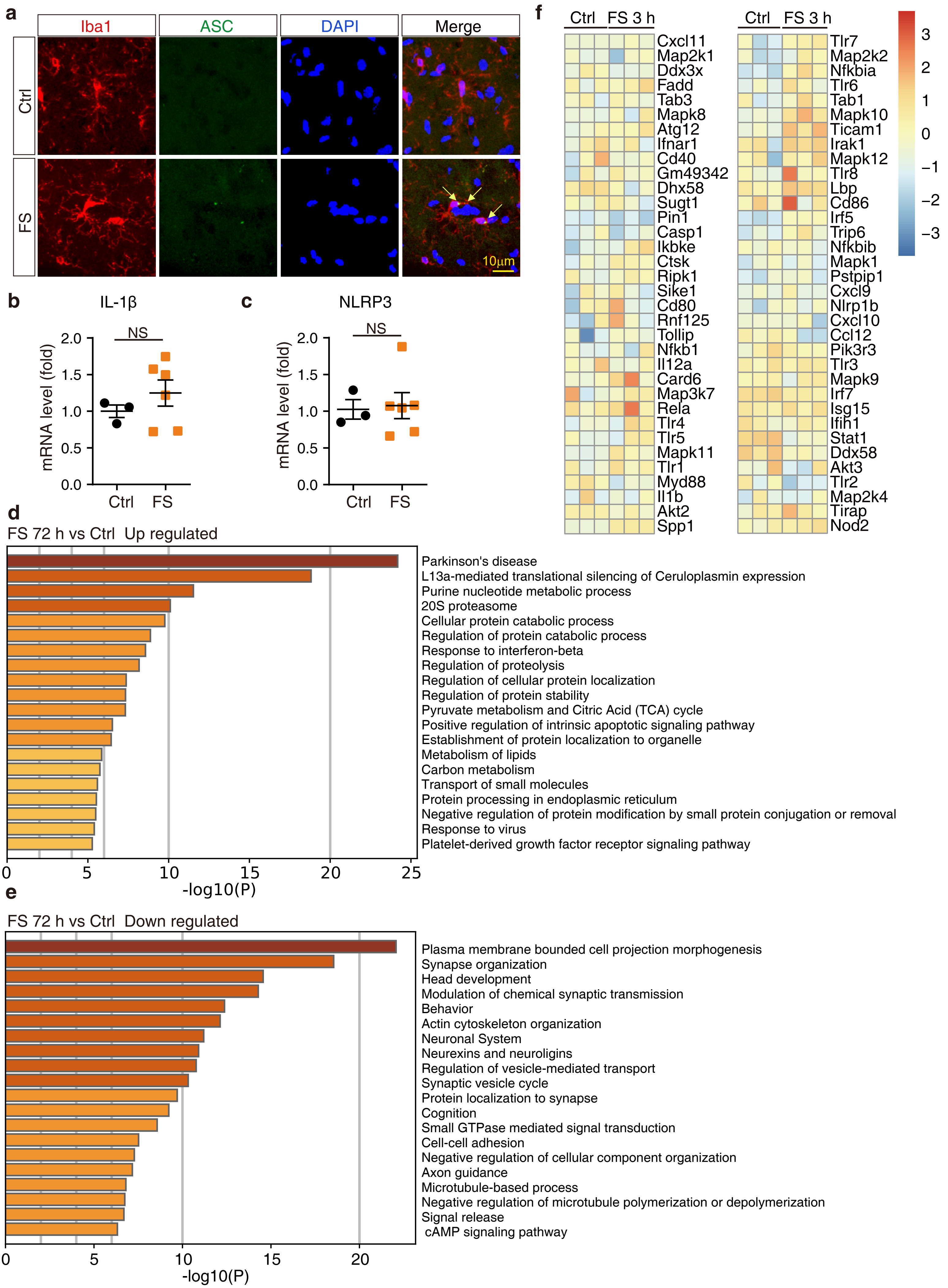
a
Iba1
ASC
DAPI
Merge
Ctrl
FS
10μm
b
IL-1β
NS
mRNA level (fold)
2.0
1.5
1.0
0.5
0.0
Ctrl
FS
c
NLRP3
NS
mRNA level (fold)
2.0
1.5
1.0
0.5
0.0
Ctrl
FS
d
FS 72 h vs Ctrl Up regulated
Parkinson's disease
L13a-mediated translational silencing of Ceruloplasmin expression
Purine nucleotide metabolic process
20S proteasome
Cellular protein catabolic process
Regulation of protein catabolic process
Response to interferon-beta
Regulation of proteolysis
Regulation of cellular protein localization
Regulation of protein stability
Pyruvate metabolism and Citric Acid (TCA) cycle
Positive regulation of intrinsic apoptotic signaling pathway
Establishment of protein localization to organelle
Metabolism of lipids
Carbon metabolism
Transport of small molecules
Protein processing in endoplasmic reticulum
Negative regulation of protein modification by small protein conjugation or removal
Response to virus
Platelet-derived growth factor receptor signaling pathway
0
5
10
15
20
25
-log10(P)
e
FS 72 h vs Ctrl Down regulated
Plasma membrane bounded cell projection morphogenesis
Synapse organization
Head development
Modulation of chemical synaptic transmission
Behavior
Actin cytoskeleton organization
Neuronal System
Neurexins and neuroligins
Regulation of vesicle-mediated transport
Synaptic vesicle cycle
Protein localization to synapse
Cognition
Small GTPase mediated signal transduction
Cell-cell adhesion
Negative regulation of cellular component organization
Axon guidance
Microtubule-based process
Negative regulation of microtubule polymerization or depolymerization
Signal release
cAMP signaling pathway
0
5
10
15
20
-log10(P)
f
Ctrl
FS 3 h
Cxcl11
Map2k1
Ddx3x
Fadd
Tab3
Mapk8
Atg12
Ifnar1
Cd40
Gm49342
Dhx58
Sugt1
Pin1
Casp1
Ikbke
Ctsk
Ripk1
Sike1
Cd80
Rnf125
Tollip
Nfkb1
Il12a
Card6
Map3k7
Rela
Tlr4
Tlr5
Mapk11
Tlr1
Myd88
Il1b
Akt2
Spp1
Ctrl
FS 3 h
Tlr7
Map2k2
Nfkbia
Tlr6
Tab1
Mapk10
Ticam1
Irak1
Mapk12
Tlr8
Lbp
Cd86
Irf5
Trip6
Nfkbib
Mapk1
Pstpip1
Cxcl9
Nlrp1b
Cxcl10
Ccl12
Pik3r3
Tlr3
Mapk9
Irf7
Isg15
Ifih1
Stat1
Ddx58
Akt3
Tlr2
Map2k4
Tirap
Nod2
3
2
1
0
−1
−2
−3

Figure S3

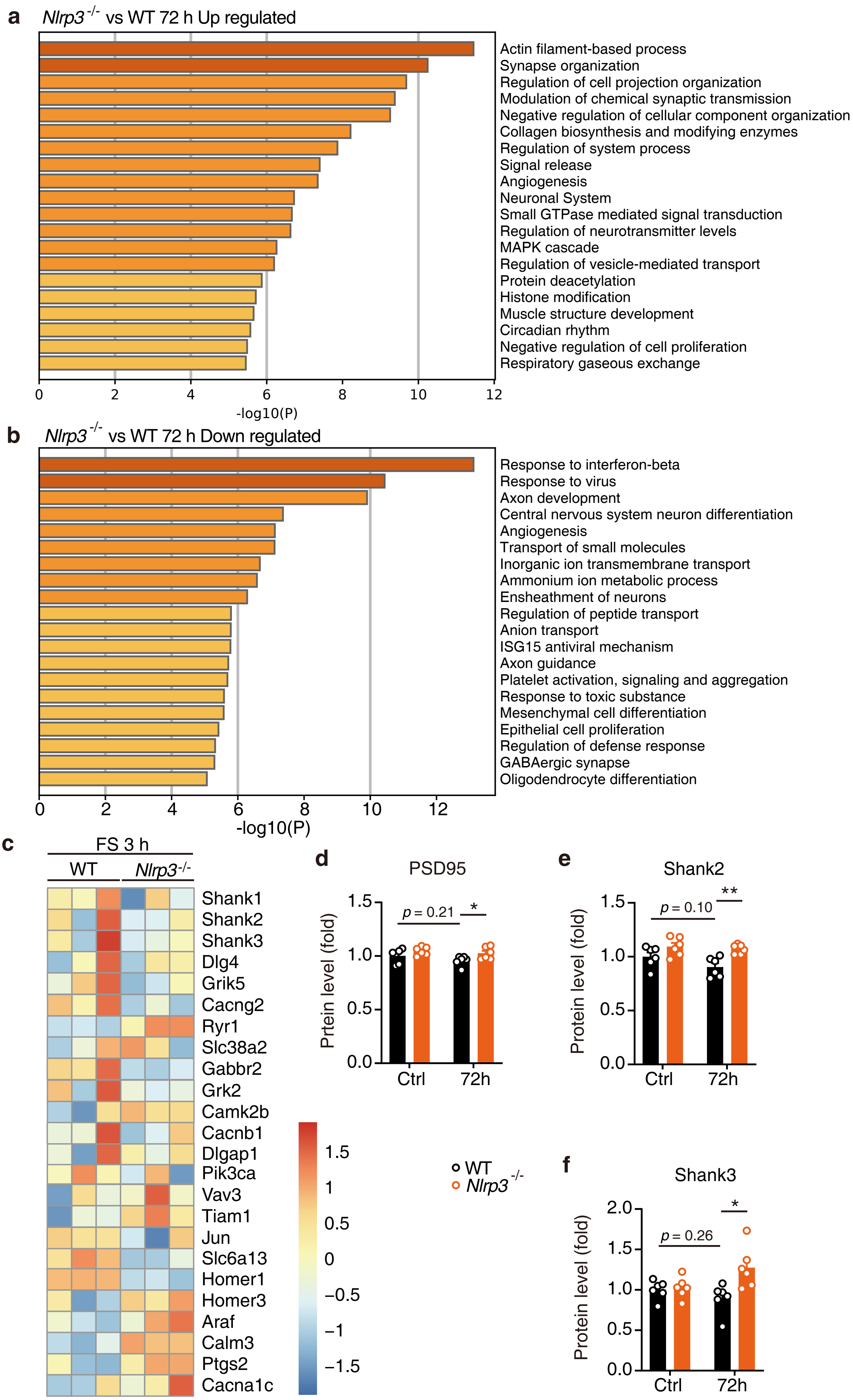
a
Nlrp3 -/- vs WT 72 h Up regulated
Actin filament-based process
Synapse organization
Regulation of cell projection organization
Modulation of chemical synaptic transmission
Negative regulation of cellular component organization
Collagen biosynthesis and modifying enzymes
Regulation of system process
Signal release
Angiogenesis
Neuronal System
Small GTPase mediated signal transduction
Regulation of neurotransmitter levels
MAPK cascade
Regulation of vesicle-mediated transport
Protein deacetylation
Histone modification
Muscle structure development
Circadian rhythm
Negative regulation of cell proliferation
Respiratory gaseous exchange
0 2 4 6 8 10 12
-log10(P)
b
Nlrp3 -/- vs WT 72 h Down regulated
Response to interferon-beta
Response to virus
Axon development
Central nervous system neuron differentiation
Angiogenesis
Transport of small molecules
Inorganic ion transmembrane transport
Ammonium ion metabolic process
Ensheathment of neurons
Regulation of peptide transport
Anion transport
ISG15 antiviral mechanism
Axon guidance
Platelet activation, signaling and aggregation
Response to toxic substance
Mesenchymal cell differentiation
Epithelial cell proliferation
Regulation of defense response
GABAergic synapse
Oligodendrocyte differentiation
0 2 4 6 8 10 12
-log10(P)
c
FS 3 h
WT
Nlrp3-/-
Shank1
Shank2
Shank3
Dlg4
Grik5
Cacng2
Ryr1
Slc38a2
Gabbr2
Grk2
Camk2b
Cacnb1
Dlgap1
Pik3ca
Vav3
Tiam1
Jun
Slc6a13
Homer1
Homer3
Araf
Calm3
Ptgs2
Cacna1c
1.5
1
0.5
0
−0.5
−1
−1.5
d
PSD95
Prtein level (fold)
1.5
1.0
0.5
0.0
p = 0.21
*
Ctrl
72h
e
Shank2
Protein level (fold)
1.5
1.0
0.5
0.0
p = 0.10
**
Ctrl
72h
WT
Nlrp3 -/-
f
Shank3
Protein level (fold)
2.0
1.5
1.0
0.5
0.0
p = 0.26
*
Ctrl
72h

Figure S4

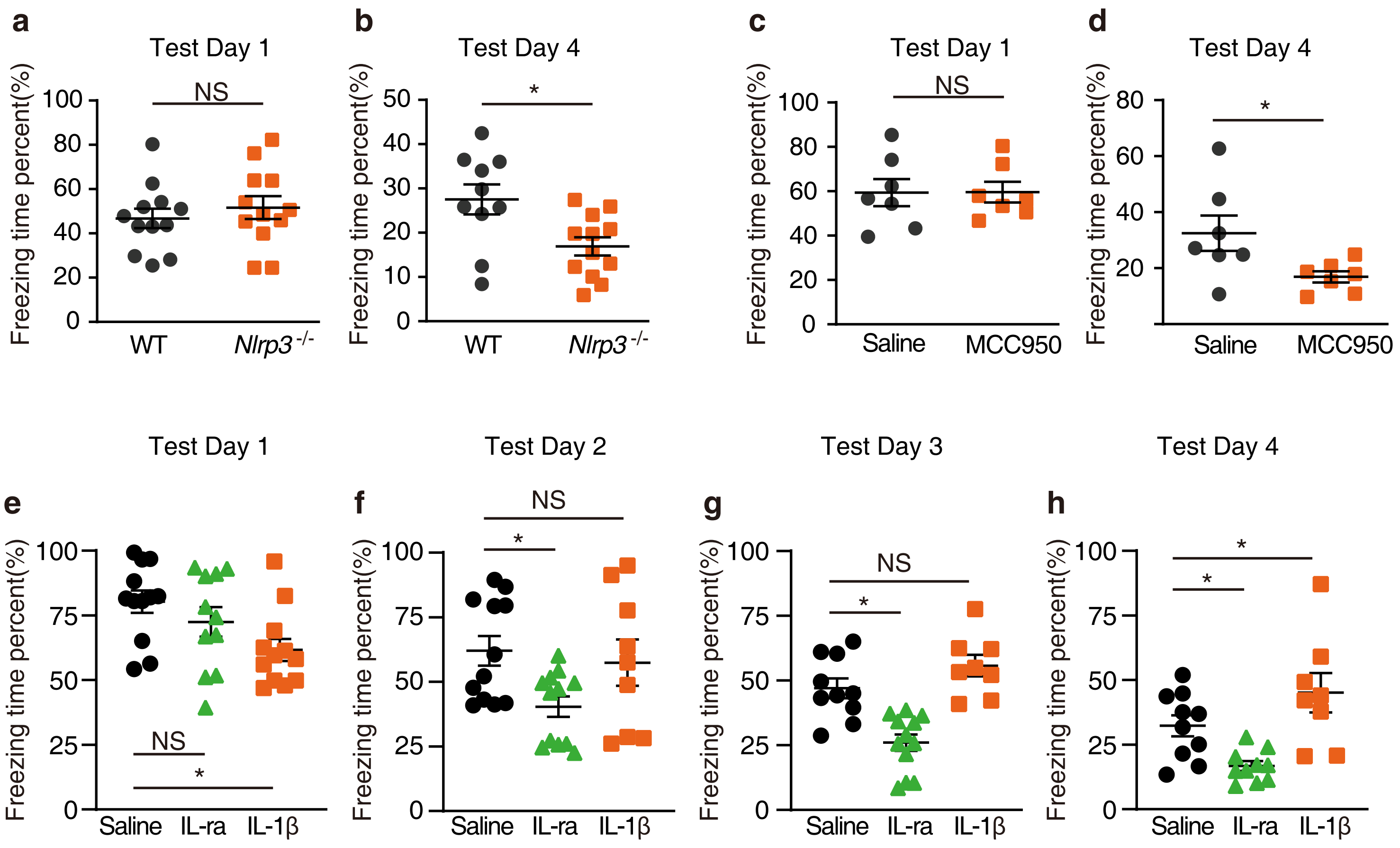
a
Test Day 1
NS
Freezing time percent(%)
100
80
60
40
20
0
WT
Nlrp3 -/-
b
Test Day 4
*
Freezing time percent(%)
50
40
30
20
10
0
WT
Nlrp3 -/-
c
Test Day 1
NS
Freezing time percent(%)
100
80
60
40
20
0
Saline
MCC950
d
Test Day 4
*
Freezing time percent(%)
80
60
40
20
Saline
MCC950
Test Day 1
Test Day 2
Test Day 3
Test Day 4
e
Freezing time percent(%)
100
75
50
25
0
NS
*
Saline
IL-ra
IL-1β
f
NS
*
Freezing time percent(%)
100
75
50
25
0
Saline
IL-ra
IL-1β
g
NS
*
Freezing time percent(%)
100
75
50
25
0
Saline
IL-ra
IL-1β
h
*
*
Freezing time percent(%)
100
75
50
25
0
Saline
IL-ra
IL-1β
